# Microinfarcts in an older population‐representative brain donor cohort (MRC CFAS): Prevalence, relation to dementia and mobility, and implications for the evaluation of cerebral Small Vessel Disease

**DOI:** 10.1111/nan.12363

**Published:** 2016-10-28

**Authors:** P. G. Ince, T. Minett, G. Forster, C. Brayne, S. B. Wharton, the Medical Research Council Cognitive Function and Ageing Neuropathology Study

**Affiliations:** ^1^ Sheffield Institute for Translational Neuroscience University of Sheffield Sheffield UK; ^2^ Institute of Public Health University of Cambridge Cambridge UK; ^3^ Department of Radiology University of Cambridge Cambridge UK

**Keywords:** microinfarct, dementia, small vessel disease, white matter lesions, lacunes, vascular risk factors, mobility, epidemiological neuropathology

## Abstract

**Introduction:**

Microinfarcts, small ischaemic foci common in ageing brain, are associated with dementia and gait dysfunction. We determined their relationship with dementia, mobility and cerebrovascular disease in an older population‐representative brain donor cohort. These data on microinfarcts were evaluated in relation to pathological assessments of clinically significant cerebral small vessel disease (SVD).

**Methods:**

Microinfarcts were assessed in the MRC Cognitive Function and Ageing Study (n = 331). Nine brain areas were staged according to the number of areas affected.

**Results:**

36% of brains showed at least 1 microinfarct. Higher cortical microinfarct stage was associated with dementia at death (OR 1.41, 95% CI 1.02; 1.96, *P* = 0.038), whilst cortical and subcortical microinfarct stages were associated with impaired mobility (OR 1.36, 95% CI 1.05–1.74; *P* 0.018) and falls (OR 1.96, 95% CI 1.11–3.43; *P* = 0.02). Adding data on microinfarcts to a definition of SVD, based on white matter lesions (WMLs), lacunes and significant arteriosclerosis, were assessed by comparing area under ROC curve (AUC) with and without microinfarcts. SVD was significantly related to dementia status with or without inclusion of microinfarcts. Modelling potential pathological definitions of SVD to predict dementia or impaired mobility indicated optimal prediction using combined assessment of WMLs, lacunes and microinfarcts.

**Conclusion:**

Cortical (dementia) and subcortical microinfarcts (impaired mobility) are related to diverse clinical outcomes. Optimal pathological assessment of significant SVD in brain ageing is achieved based on WMLs, lacunes and microinfarcts and may not require subjective assessment of the extent and severity of arteriosclerosis.

## Introduction

In neuropathological practice the term ‘microinfarct’ is used to describe CNS lesions of presumed ischaemic origin that are only detected by microscopic examination 1. Such lesions are a common finding at autopsy in older individuals 2. They are typically considered to measure less than 1 mm in maximum diameter, although no consensus definition exists, and are frequently much smaller. Anatomically they are encountered in the cerebral cortex, white matter, deep grey nuclei, midbrain, brainstem and cerebellum. They are morphologically and pathogenetically distinct from periarterial or periarteriolar space expansion. They may include a small area of cavitation but frequently do not. Noncavitated lesions are recognizable as areas of neuropil or white matter attenuation associated with gliosis and loss of neuronal elements. They commonly contain a variable number of reactive astrocytes, amoeboid microglia and siderophages and the pathogenesis of microinfarction remains unclear. In the context of cerebral amyloid angiopathy (CAA) they occur in the cerebral cortex, particularly if there are associated features of severe or complicated CAA (microaneurysms, perivascular inflammation, etc.). When they do not occur as part of the parenchymal sequelae of CAA it is assumed that other factors lead to occlusion, or critically low flow, in small distal arterioles. Such factors likely include intrinsic small vessel degenerative changes (possibly related to systemic hypertension). As such, microinfarcts can reasonably be included within the spectrum of vascular lesions encompassed by the term ‘small vessel disease’ (SVD).

Microinfarcts are reported to be associated with a number of clinical and pathological observations in older people. Virtually all such studies to date are based on post‐mortem associations because microinfarcts, as defined above, are not demonstrated at magnetic field strengths (i.e. 3Tesla and below) used currently in routine clinical magnetic resonance imaging (MRI) 3. The term has, however, been applied to imaging studies, from the earliest use of computed tomography brain scanning, in the context of lesions of several millimetres in diameter so that there is a problematic inconsistency in terminology between pathologists and radiologists 4, 5. They are reported to relate to the presence of macroscopic cerebral infarcts (both lacunes and larger infarcts) and atherosclerosis of cerebral arteries 6, 7. They are associated with cerebral atrophy, dementia and also cognitive decline in nondemented people 8, 9, 10, 11. Larger numbers of microinfarcts in an individual may increase the risk of dementia. They are additionally associated with an increased risk of depression in older people, especially if the burden of disease is subcortical 12. A large population‐representative study (Honolulu Asia Ageing Study: HAAS) has suggested that microinfarcts have a predominant significance in the pathological evaluation of small vessel vascular lesions contributing to cognitive decline and dementia 10.

MRC CFAS, a longitudinal, population‐representative cohort study of cognition and frailty in older people in the UK, has contributed substantially to the emerging consensus that vascular pathology is a major correlate of cognitive decline and dementia in old people 13, 14. Our previous analyses have shown that the effect of vascular disease on cognition is primarily mediated through SVD rather than the concept of multi‐infarct dementia that emerged from studies published in the 1970s 15, 16. SVD is considered to be the pathological basis of the clinical diagnosis of Subcortical Ischaemic Encephalopathy in most cases 1. CFAS studies have previously used a modification of the Consortium to Establish a Registry for Alzheimer's Disease (CERAD) neuropathology protocol to record and score pathological changes in the brains donated by respondents and their relatives. The CFAS working definition of SVD has been based on the presence of any one of: moderate or severe arteriosclerosis of parenchymal vessels; lacunar infarcts (i.e. macroscopically identified old ischaemic lesions <1 cm in diameter); severe white matter lesions (WML; corresponding to Schelten's scores 5 and 6); and microinfarcts 13. All these evaluations include compromises based on the methods available and which are discussed below. However, the CERAD protocol used was drafted prior to the emergence of microinfarcts as an important pathology so that this type of lesion did not have a specific data field captured within the CFAS pathology data proforma 17. The core CERAD data set was accumulated from multiple centres involving evaluations made by a number of different pathologists. Inter‐rater reliability has been established previously for core CERAD data items (atrophy, neuritic plaques, tangles, Lewy bodies, etc.) but not for microinfarcts 14. Some pathologists recorded microinfarcts as free text items but there was no systematic approach and these data are previously incomplete.

In this study the brain donations to three of the six CFAS centres were re‐evaluated by a single neuropathologist (SBW) to record the location and number of microinfarcts in all the blocks used for CERAD evaluations. These new data have allowed us to address a number of important questions.

How are microinfarcts associated, in this population representative cohort, with other types of small and large vessel ischaemia? Does the addition of complete data on microinfarcts alter previous conclusions about SVD published by CFAS, in particular by increasing the ascertainment of an SVD diagnosis, and in terms of the impact on pathological correlates of dementia? Do the findings and analyses offer useful insights into operationalizing the pathological evaluation of SVD?

## Material and methods

### Assessment of small vascular lesions

**Figure 1:**
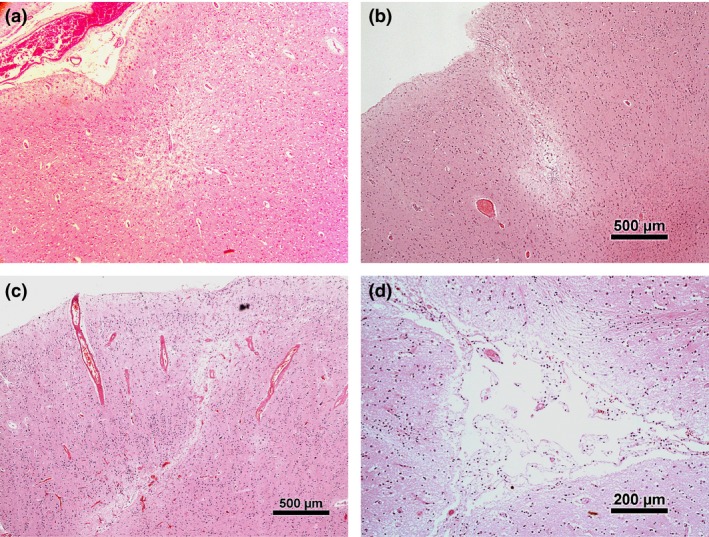
Photomicrographs of cortical and subcortical microinfarcts/microscopic lesions. Microinfarcts (block arrows) typically appear as small localized areas of cortical rarefaction frequently in clear association with a small penetrating artery (arrow head) (**a,b**). In (**c**), the microinfarct has a linear configuration extending towards the cortical surface (block arrows). There is eosinophilia and vessel wall thickening of the penetrating arteries in the adjacent cortex typical of amyloid angiopathy (arrows). Basal ganglia lesions frequently include a cystic component with variable attenuation of the contiguous neuropil (arrowhead) (**d**). [Colour figure can be viewed at wileyonlinelibrary.com]

All brain donations (n = 331) corresponding to CFAS data release 4.0 from 3 CFAS Centres (Cambridge, Newcastle upon Tyne, Nottingham) were examined for this study. These brains were prepared for archiving as previously described 14. One cerebral hemisphere was dissected fresh (Cambridge, Newcastle upon Tyne) or partially sampled (Nottingham) for rapid freezing and storage. The remaining tissues were formalin fixed and dissected and prepared for macroscopic and microscopic brain examination using standard methods. For this study, microinfarcts were detected using screening with a low power objective (×4 objective) on haematoxylin and eosin (H&E) stained sections of formalin‐fixed paraffin‐embedded tissue. All lesions observed were then confirmed at higher power magnification. A microinfarct diagnosis was confirmed for all lesions smaller than 1 mm in diameter. Sections from the following nine brain regions were examined for microinfarcts: frontal cortex, temporal cortex, parietal cortex, occipital cortex, cingulate cortex, insular cortex, hippocampus (level of lateral geniculate body), entorhinal cortex and amygdala, cerebellar cortex. Sections from the following four subcortical brain regions were examined for microscopic subcortical lesions: basal ganglia, midbrain, pons and medulla. Examples of lesions identified in cortical and subcortical sites are shown in Figure 1.

An indication of the severity and anatomical dispersion of lesions was recorded as the number of regions in which microinfarcts and subcortical lesions were present in each case. For the analysis in this study, we have used the word ‘stage’ to encompass the number of regions affected although we do not imply any hierarchic element to the anatomical dispersion or severity of microinfarcts.

### Other small vessel disease

Existing CFAS data were used to define SVD in the absence of either microinfarcts/subcortical lesions detected in the review conducted for this analysis as described above, or any previous microinfarct data that had been recorded. Details of the definitions and techniques used to detect these lesions are described previously. Briefly, severe or moderate arteriosclerosis was evaluated by microscopy in H&E sections as thickened vessel profiles with variable hyalinization of the muscle media and loss of smooth muscle nuclei. Lacunes were cystic lesions measuring less than 1 cm in diameter that were observed at brain dissection by macroscopic inspection. WMLs were detected by MRI scanning of 3 formalin fixed, 1 cm thick, coronal cerebral hemisphere slices as previously described. Significant WML, in terms of a CFAS diagnosis of SVD, corresponds to the presence of WML at modified Schelten's scale scores of 5 or 6 in any of this sample. Scheltens’ score 5 implies the presence of at least one area of deep hemispheric white matter T2 signal attenuation greater than 11 mm in any diameter. Scheltens’ score 6 refers to the presence of larger and more confluent areas of MRI white matter attenuation. Previous work in CFAS including this cohort of brain donors has shown that scores of 5 and 6 are significantly associated with risk of dementia 15.

### Assessment of CAA

Data for CAA were available from existing CFAS data collected by neuropathologists blind to clinical data using immunohistochemical or tinctorial methods. The severity of meningeal and parenchymal CAA was scored semi‐quantitatively according to the CERAD protocol, as either ‘none’, ‘mild’, ‘moderate’ or ‘severe’, in the hippocampal, entorhinal and neocortical regions. A final semi‐quantitative score was obtained by calculating the maximum score across these areas. These scores were simplified for analysis: ‘severe’ was merged with ‘moderate’, and ‘none’ was merged with ‘mild’.

### Assessment of clinical and other data

All CFAS brain donors underwent longitudinal assessments in life using a standard interview battery. Existing data relating to clinical diagnoses and self‐reported risk factors were available including dementia status at death (based on data from the last assessment before death and a Retrospective Informant Interview [RINI]), and any self‐reported medical history of angina, heart attack, stroke, systemic hypertension or diabetes mellitus (DM). The ApoEε4 status for each brain donor was known from previous genetic analyses. In the absence of formal gait‐analysis data, a mobility score was created by combining CFAS interview questions regarding mobility and tendency to fall, and based on the last interview before death. A semi‐quantitative score of 0 was assigned if the participant was ambulant, score 1 if there were recent falls (defined as more than one fall since previous interview), and score 3 if the participant was chair or bed bound.

### Statistical analysis and modelling of diagnostic protocols

Logistic regression analyses were used to verify the relationship between the presence of dementia and vascular lesions. To study the relationship between mobility scores and microinfarct stages, and between microinfarct stages and WML scores, ordinal logistic regression analyses were used. Because microinfarct stages are counts of regions containing lesions, and were over‐dispersed, negative binomial regression was used when assessing risk factors for microinfarct stages. The effect of adding data on microinfarcts into the definition of SVD was assessed by comparing the logistic regression models with and without microinfarcts based on area under receiver‐operating characteristic curve (AUC). All tests were two‐tailed. Confidence intervals (95% CI) were calculated for the odds ratio (OR), incidence rate ratio (IRR), β and AUC. A *P*‐value of less than 0.05 indicated statistical significance. Statistical analyses were performed using statistical package STATA, version 12.

## Results

### Characteristics of the cohort regarding dementia status

The demographic and pathological details of the cohort are presented in Table 1. In 9% of respondents the dementia status was not clear from the data available from respondent interviews and the RINI. Such cases are not included in analyses related to dementia.

**Table 1:** Demographic, cognitive and mobility profile according to clinical dementia status

	No dementia (n = 126)	Dementia (n = 174)	Not determined (n = 31)
Women^a^	63 (50)	119 (68)	16 (52)
Age at death^b^	84 (76–89)	89 (84–93)	86 (83–89)
Years since last cognitive assessment^b^	1.2 (0.7–2.0)	2.3 (1.0–3.7)	2.1 (1.8–3.4)
MMSE at last assessment^b^	26 (23–28)	14 (8–20)	27 (22–28)
Years since last mobility assessment^b^	1.3 (0.7–2.2)	3.1 (1.8–3.4)	0 (0)
Mobility score at last assessment^b^	0 (0–1)	0 (0–0)	0 (0–1)

a n (%).

b Median (inter quartile range).

### Relationship between vascular neuropathology and clinical status

Overall 36% of brains contained at least one microinfarct. The frequency of microinfarcts in cortical and subcortical regions according to dementia status is shown in Table 2. To explore whether microinfarcts were related to dementia, logistic regression analyses were performed with dementia status as dependent variable and microinfarct stages as independent variable controlling for sex and age at death (Table 3). More regions with cortical microinfarcts were significantly associated with clinical dementia but neither subcortical lesion burden nor total burden (combined cortical and subcortical) showed a significant relationship with dementia status.

**Table 2:** Microinfarct distribution according to clinical dementia status

Region^a^	No dementia (n = 126)	Dementia (n = 174)	Not determined (n = 31)
Cortical microinfarcts^b^	0 (0; 3)	0 (0; 6)	0 (0; 6)
Frontal cortex^a^	5 (4.0)	15 (8.6)	3 (9.7)
Temporal cortex^a^	1 (0.8)	9 (5.2)	1 (3.2)
Parietal cortex^a^	4 (3.2)	10 (5.8)	1 (3.2)
Occipital cortex^a^	12 (9.5)	13 (7.5)	5 (16.1)
Cingulate cortex^a^	0	4 (2.3)	0
Insular cortex^a^	0	1 (0.6)	1 (3.2)
Hippocampal cortex^a^	5 (4.0)	6 (3.5)	1 (3.2)
Entorhinal cortex^a^	0	11 (6.3)	1 (3.2)
Cerebellar cortex^a^	6 (4.8)	6 (3.5)	1 (3.2)
Subcortical lacunar infarcts^b^	0 (0; 2)	0 (0; 3)	0 (0; 2)
Basal ganglia^a^	13 (10.3)	24 (13.8)	4 (12.9)
Midbrain^a^	2 (1.6)	1 (0.6)	1 (3.2)
Pons^a^	4 (3.2)	7 (4.0)	1 (3.2)
Medulla^a^	1 (0.8)	1 (0.6)	0
Total microinfarcts^b^	0 (0; 4)	0 (0; 6)	0 (0; 7)

a Number of brains with one or more microinfarct per region (%).

b Median number of affected regions (minimum; maximum).

**Table 3:** Logistic regression analysis of the relationship between microinfarct stages and clinical dementia status or mobility

Stages	Dementia status			Mobility score		
	OR	95% CI (OR)	*P*	OR	95% CI (OR)	*P*
Cortical	1.41	(1.02; 1.96)	0.038	1.36	(1.05; 1.74)	0.018
Subcortical	1.05	(0.99; 2.72)	0.846	1.96	(1.11; 3.43	0.020
Total	1.30	(0.99; 1.69)	0.056	1.39	(1.44; 1.74)	0.004

Using the mobility scores generated from the interview data as the dependent variable in ordinal logistic regression analysis, and microinfarct stages as independent variables, higher microinfarct stages in cortical, subcortical and total regions were significantly related to impaired mobility and falls. The analysis was controlled by age of death, sex, education and gap between last mobility assessment and death (Table 3).

### Relationship between vascular neuropathology and risk factors

The prevalence of self‐reported hypertension (HTN) was 39%, Diabetes mellitus (DM) 11%, angina 19%, myocardial infarct (MI) 12% and cerebrovascular accident (CVA) 15% in this cohort of CFAS respondents. Twenty‐three percent of the respondents had at least one APOEε4 allele. Moderate or severe CAA was present in 22% of the brains. Negative binomial regression was used to investigate possible risk factors for microinfarction. Microinfarcts stages were selected as dependent variables and angina, CVA, DM, HTN, MI, presence of at least one APOEε4 allele, and moderate or severe CAA, were selected as independent variables. All analyses were controlled by age at death and sex (Table 4). There was a significant relationship between the presence of microinfarcts in both cortical and subcortical locations and a reported history of CVA. A relationship with hypertension was demonstrated for total lesions although it was not significantly associated when analysis is performed separately for cortical and subcortical regions.

**Table 4:** Negative binomial regression analyses on the relationship between cardiovascular risk factors and microinfarcts

Risk factors	IRR	95% CI (IRR)	*P*	IRR	95% CI (IRR)	*P*	IRR	95% CI (IRR)	*P*
		Cortical microinfarct			Subcortical lesion			Total microinfarct	
Angina	1.53	(0.90; 2.60)	0.110	0.96	(0.47; 1.94)	0.910	1.35	(0.86; 2.13)	0.200
Stroke	2.74	(1.62; 4.64)	<0.001	1.91	(1.02; 3.59)	0.040	2.47	(1.58; 3.87)	<0.001
DM	0.75	(0.34; 1.63)	0.460	0.71	(0.25; 2.02)	0.520	0.72	(0.37; 1.40)	0.330
HTN	1.42	(0.89; 2.26)	0.140	1.56	(0.90; 2.69)	0.110	1.47	(1.00; 2.18)	0.050
MI	1.42	(0.75; 2.67)	0.280	1.01	(0.45; 2.29)	0.980	1.29	(0.75; 2.22)	0.360
ε4	0.57	(0.31; 1.05)	0.070	1.35	(0.68; 2.67)	0.400	0.78	(0.48; 1.27)	0.310
CAA	1.47	(0.89; 2.44)	0.130	1.25	(0.68; 2.29)	0.470	1.39	(0.90; 2.13)	0.130

DM, diabetes mellitus; HTN, systemic hypertension; MI, myocardial infarction; ε4, 1 or 2 Apolipoprotein E ε4 alleles; CAA, cerebral amyloid angiopathy.

### Relationship between vascular neuropathology and MRI WML

MRI evaluation of WML using Schelten's modified score in this cohort showed a deep WML median score of 3 (range 0–6) and a periventricular (PV) WML median score of 2 (range 0–3). To explore a relationship between MRI WML and microinfarcts ordinal logistic regression analyses were used with deep WML and PVWML scores as dependent variables. Microinfarct stages were selected as independent variable, and all analyses were controlled by age of death and sex (Table 5).

**Table 5:** Ordinal logistic regression analyses on the relationship between microinfarct stage and MRI WML

Stages	Deep WML			Periventricular WML		
	OR	95% CI (OR)	*P*	OR	95% CI (OR)	*P*
Cortical	1.31	(1.01; 1.70)	0.044	1.17	(0.89; 1.54)	0.264
Subcortical	1.35	(0.83; 2.19)	0.228	1.80	(1.09; 2.97)	0.022
Total	1.30	(1.04; 1.62)	0.023	1.27	(1.01; 1.61)	0.045

Cortical microinfarcts in more regions was significantly related to higher deep WML scores.

### Effect of the inclusion of microinfarcts as a component of SVD in relation to dementia status

Among the 300 participants in whom clinical dementia status was established, SVD was present in 55 participants without dementia and 109 with dementia. The addition of microinfarcts into the operational definition of SVD identified 11 additional respondents with SVD: seven of these had clinical dementia and four did not. To investigate the effect of adding data on microinfarcts into the definition of SVD in relation to clinical dementia status, we compared the area under ROC curve (AUC) for two logistic regression models: Model 1 used the previously defined CFAS SVD status without microinfarct data; Model 2 incorporated microinfarct data into the previously defined CFAS SVD status. Both models used clinical dementia status as dependent variable and SVD as independent variable, and were controlled for age of death and sex. SVD was significantly related to dementia status in both models but the two AUC were not significantly different.

### Analysis of components of the SVD diagnosis in the design of diagnostic protocols for the pathological evaluation of SVD

Since the effect of microinfarcts on dementia status was modest we additionally explored the contribution of each component used in the operational definition of significant SVD and both dementia status and mobility. The contribution of the presence of each SVD component (lacunes, cortical microinfarcts, subcortical lesions, moderate or severe arteriolar sclerosis and WML) and their relationship with dementia and mobility status, controlled by sex and age, is demonstrated in Table 6. When analysing the contribution of each SVD component independently, only white matter pallor was significantly related to dementia status, independently of age and sex. The only single lesion type significantly associated with mobility was microinfarction. Analysis of the effect of combinations of lesion types suggests that excluding arteriosclerosis from the working definition of SVD has a very limited impact on the relationship with dementia or with mobility. Assessment of arteriosclerosis is based on distinguishing relative severity and has not been operationalized or assessed by inter‐rater reliability studies. A diagnostic protocol based exclusively on the presence or absence of the lesions considered to result from SVD, rather than on the subjective assessment of vessels themselves, is likely to be a more robust approach for pathological studies.

**Table 6:** Logistic regression analysis of the relationship between the individual components, and possible combinations of components, used to define SVD and clinical outcomes. *p* values in bold type indicate statistically significant association with clinical outcome

SVD definition	Total (n)	Dementia				Impaired mobility				
		With dementia (n)	OR	95% CI (OR)	*P*	Frequent falls (n)	Chair/bed (n)	OR	95% CI (OR)	*P*
A	68	36	0.86	(0.46; 1.60)	0.630	17	5	1.61	(0.88; 2.95)	0.119
L	63	35	1.45	(0.75; 2.82)	0.270	14	6	1.76	(0.95; 3.26)	0.075
M	118	69	1.48	(0.88; 2.48)	0.143	28	9	2.07	(1.21; 3.52)	**0.008**
W	137	86	1.76	(1.06; 2.93)	**0.030**	24	8	1.01	(0.59; 1.73)	0.966
MA	152	86	1.24	(0.75; 2.03)	0.404	35	12	2.26	(1.32; 3.85)	**0.003**
MW	187	112	1.95	(1.18; 3.23)	**0.010**	35	11	1.18	(0.69; 2.01)	0.543
WA	163	100	1.85	(1.12; 3.06)	**0.016**	28	11	1.06	(0.63; 1.80)	0.827
LA	99	53	1.10	(0.63; 1.90)	0.742	22	7	1.44	(0.84; 2.47)	0.190
LM	157	91	1.58	(0.96; 2.60)	0.072	35	13	2.17	(1.28; 3.68)	**0.004**
LW	167	101	1.89	(1.15; 3.12)	**0.013**	32	12	1.37	(0.81; 2.31)	0.237
LMA	173	99	1.46	(0.89; 2.40)	0.132	38	14	2.18	(1.28; 3.74)	**0.004**
LMW	210	124	2.12	(1.26; 3.58)	**0.005**	40	14	1.38	(0.79; 2.40)	0.252
LWA	179	108	1.88	(1.14; 3.11)	**0.014**	32	13	1.20	(0.71; 2.02)	0.505
WMA	203	120	1.99	(1.19; 3.32)	**0.009**	37	13	1.19	(0.69; 2.05)	0.525
WMLA	217	128	2.13	(1.25; 3.62)	**0.005**	40	15	1.31	(0.75; 2.30)	0.339

For dementia all models including white matter lesions are significantly predictive; For impaired mobility only models including subcortical microinfarcts are significantly predictive.

A, moderate or severe arteriosclerosis; L, lacunes; M, microinfarcts; W, deep white matter lesions.

## Discussion

The analysis confirms that microinfarction is a common finding in an unselected, population‐representative, cohort of older brain donors in the UK among whom more than 75% were aged over 80 years at death. More than one‐third of the cohort had at least one lesion. Our data analysis supports the hypothesis that such lesions are clinically relevant in terms of the risk of dementia and of impaired mobility and falling in old age, and thus we conclude that microinfarcts significantly contribute to physical and mental frailty in the elderly. These data are very similar to those reported in a large community‐based sample (Religious Orders Study) in which 30% of brain donors had microinfarcts among whom 45% did not have any macroscopic infarction 18.

The burden of microinfarcts is associated with other manifestations of cerebrovascular pathology 2, 18. At the present time the pathophysiological mechanisms linking these subtypes of vascular pathology are poorly understood. We demonstrate an association both with a clinical history of stroke and with hypertension. Hypertension is certainly considered to be a major underlying risk factor for cerebral SVD but clinical stroke is diverse both in terms of ischaemic and haemorrhagic pathophysiology and, for ischaemic stroke, in terms of the contributions of intrinsic cerebrovascular disease compared to carotid or cardiac microembolization 1. Within the constellation of pathologies that we have combined into a global concept of SVD the underlying mechanisms that cause microinfarcts, white matter attenuation and lacunes are not precisely defined. Other work has suggested that cerebral atherosclerosis is linked with microinfarction, whereas arteriosclerosis is more linked to lacunes. These findings potentially imply a role for artery‐to‐artery microembolization as a significant pathogenetic event 7. There is further supportive evidence based on an increase in haemostatic biomarkers in people with microinfarction 19. There are significant limitations on the utility of human post‐mortem‐based observations in determining the underlying mechanisms that give rise to WMLs, lacunes, microbleeds and microinfarcts so that the development of preclinical models is an urgent priority to accelerate effective translational research towards therapies with the potential to modify these different types of tissue injury.

Our working definition of SVD conflates a measure of blood vessel damage (severity of arteriosclerosis) with several pathologies assumed to represent parenchymal sequelae of presumed acute (microinfarcts, lacunes) and chronic (WMLs) ischaemia. We have used multivariate analysis to try and tease apart the impact of these various types of lesion on clinical outcomes and the association with risk factors but acknowledge that there are likely to be major common underlying mechanisms so that they are not entirely independent.

There is no established consensus on the evaluation of lesions that fall within the spectrum of SVD 20, 21, 22. Despite this a number of studies, including MRC CFAS, have shown that SVD is a more important risk factor for cognitive decline and dementia than large ischaemic lesions 15. A single report has addressed the implications of encountering microinfarcts in the very limited sample of brain tissue that is routinely evaluated by microscopy in modern neuropathology practice for the diagnosis of dementing disorders. This study calculated that if 1 microinfarct is present in any of the sections examined from nine routinely sampled brain regions (each section likely to be ~4–6 cm^2^ in surface area and 4–8 μm thick, i.e. up to 0.5 mm^3^ in volume) this likely corresponds to around 550–1100 such lesions throughout the whole brain 23. There is therefore an important issue to resolve in terms of how to operationalize the evaluation of microinfarcts, and how to assimilate such data into the wider evaluation and definition of SVD. In the basal ganglia small parenchymal lesions are especially problematic to classify. The possibility of three types of macroscopic lacune has been proposed (true lacunar infarcts, enlarged perivascular spaces, small apoplectic cysts) and all three are common in the deep grey nuclei 24. Furthermore, there appears to be some continuity between microscopic foci and those visible to the naked eye. For this reason microinfarcts and other microscopic ischaemic vascular lesions in this region were grouped together as ‘subcortical lesions’ for the purposes of this study if they were not previously detected by gross examination as lacunes or ètat cribré.

In view of the above considerations, we have examined our data to see if the assertion arising from the Honolulu Asian Ageing Study, that microinfarcts are of particular significance as predictors of clinically expressed SVD, is justified in this cohort 10. We show that the inclusion of microinfarcts does modestly increase the number of brains examined to which a diagnosis of SVD can be assigned using our criteria. However, this added individuals both with and without dementia, and the overall impact in terms of the predictive power of the SVD diagnosis was not significantly changed.

We therefore propose that a pathological concept of SVD based on a constellation of lesion types remains the most valid approach to capturing this pathological state for clinicopathological correlation and to study disease mechanisms in post‐mortem brain samples. Because WML remain an important lesion type in this analysis, and it is necessary to stratify cases by severity and lesion burden, it is reasonable to assert that neuropathological protocols that do not adequately assess WML are incomplete in terms of assessing SVD brain pathology in older people. This is most conveniently and efficiently achieved using post‐mortem MRI which is presently not widely available to neuropathology laboratories 25. We also raise the possibility that including the direct evaluation of arteriosclerosis may not be an essential element of the pathological constellation of SVD. Including arteriosclerosis requires a subjective evaluation of severity and may not be so robust to interobserver agreement. In contrast, the presence of microinfarction, lacunar infarction and extensive WMLs as demonstrated by MRI are largely categorical measures.
